# Supplementary material for: Effect of postural threat on motor control in people with and without low back pain
Source: PLoS One. 2023 Mar 27;18(3):e0280607. doi: 10.1371/journal.pone.0280607 (PMC10042370; doi:10.1371/journal.pone.0280607)
Supplement: S1 File — (DOCX) [file pone.0280607.s001.docx]

# Expected Back Strain Scale (EBS-scale)^[[1]](#footnote-1)^


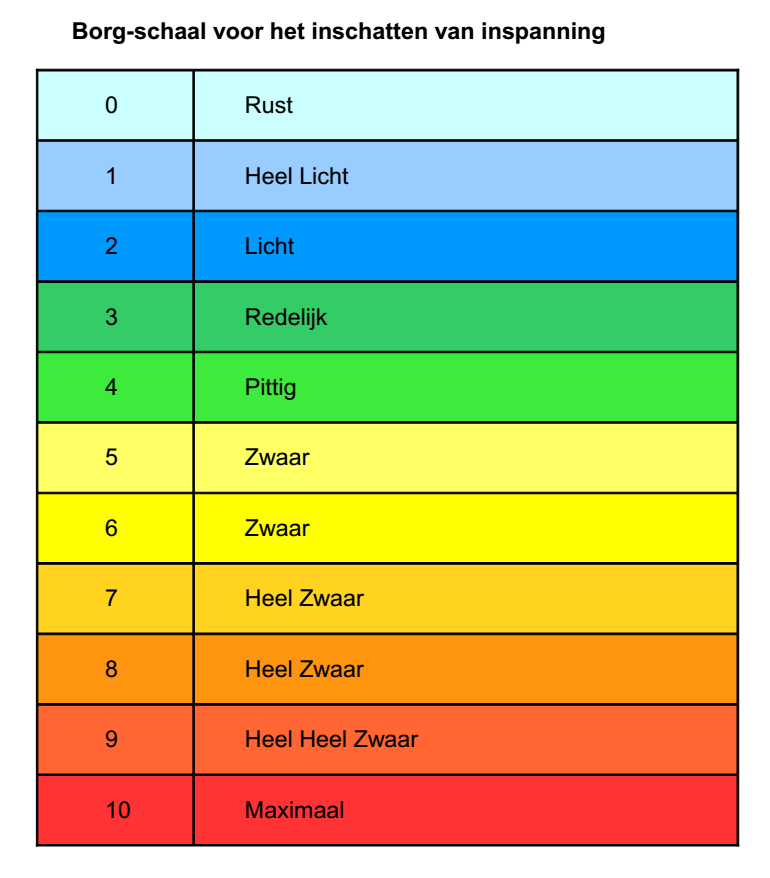
Hoe belastend denk je dat dit gaat zijn voor je rug? (How demanding will this task be for your back?)

1. Based on the colour marked Borg RPE-scale: Borg GA. Psychophysical bases of perceived exertion. Medicine and science in sports and exercise. 1982;14(5):377-381. [↑](#footnote-ref-1)
