# Supplementary material for: Effect of postural threat on motor control in people with and without low back pain
Source: PLoS One. 2023 Mar 27;18(3):e0280607. doi: 10.1371/journal.pone.0280607 (PMC10042370; doi:10.1371/journal.pone.0280607)
Supplement: Attachment — Submitted filename: Response to Reviewers5.docx [file pone.0280607.s002.docx]

Dear Reviewers,

Thank you for your constructive comments. The changes made to the manuscript in response to your comments have been copied in the rebuttal below. We have since made our data available through the Open Science Framework (OSF) and the link is referenced in the paper.

Yours sincerely,

Meta (M.H.) Wildenbeest

5. Review Comments to the Author

Please use the space provided to explain your answers to the questions above. You may also include additional comments for the author, including concerns about dual publication, research ethics, or publication ethics. (Please upload your review as an attachment if it exceeds 20,000 characters)

Reviewer #1: The manuscript “Effect of postural threat on motor control in people with and without low back pain” examines the effect of a perceived postural threat on lumbar spine kinematics in participants with and without low back pain. The manuscript is very well written and simple to follow. The data presented are valuable and the experiment itself is novel and appears to be a valid approach for inducing a perceived postural threat without requiring an actual perturbation. I would suggest, however, that the manuscript needs to be rewritten with a more conservative interpretation of the interaction effects that were observed between LBP status and the dichotomized high/low split for expected back strain (EBS).

I believe that the authors have interpretated of the main effect of ‘threat’ correctly. The data clearly demonstrate that the experimentally induced perception of a postural threat increases the temporal and spatial variability, and decreases the dynamic stability, of the lumbar movement pattern during the seated reaching task, independent of the experimental group (LBP vs. control) and the strain on the back that the participants expected from the task (EBS score).

The data also show interaction effects between group and EBS for some kinematic variables assessing movement variability. For these data, however, I do not agree fully with the authors’ interpretation that this is evidence of greater movement variability in participants with both LBP and high EBS than in the other three subgroups, regardless of the threat.

1. For the MeanSD variables in axial-rotation and lateral-bending, the authors’ interpretation has some merit. However, low EBS also appears to be associated with more variability in the control group. Because of this, as well as the limited amount of movement about these axes and the absence of any significant interaction effects for the primary axis of motion, I would be very hesitant to interpret these interactions without a model that accounts for the opposite effects of EBS on the control and LBP groups. Some explanation would also be warranted for why these effects did not suffer from the same “ceiling effect” that is alluded to as an explanation for why no main effect of threat was found in these off-axis movement data (bottom of page 14). I would expect any centrally-mediated effect be most evident in the primary axis movement (unless a less flexible motor control system is not capable exerting the same level of control in all movement axes).

Low EBS present in the healthy group was not associated with increased variability. Our conclusion is based on the post-hoc comparisons, in which only the group high EBS with LBP differed from all others. In short, we don’t think that our model should account for opposite, non-significant effects.

Indeed, we only found a between-subject interaction effect for the non-primary axes of motion. An increase in variability only in the non-primary axes with low back pain has also been observed in other studies:

- Decreased mean lateral bending stiffness during repetitive trunk flexion/extension (F-ratio=4.403,p=0.037) (1).
- A more variable coordination in the frontal plane, during walking (2)

We are not sure why these between-subject effects are observed for movement around the secondary axes. One might suggest that task constraints limit the scope to vary movements around the primary axis and an alternative explanation could be that an increased feedback gain (in the high EBS and LBP group) increases movement variability around the secondary axes more, because intrinsic stiffness is lower around these axes. However, the effect of threat was observed for movement around the primary axis, which would contradict both of these explanations.

With respect to the ceiling effect, this was invoked to explain why no effect of threat was observed around the secondary axes. The idea being that part of the group (LBP + high EBS) already had increased variability in the reference condition. In that group no increased variability around the primary axis in the reference condition was found. We note here that, although threat and group did not significantly interact, the back-healthy participants with low EBS showed increased off-axis variability during the postural threat condition (p = 0.03 for twisting and p = 0.073 for lateral bending), but the LBP participants with high EBS did not, which caused the absence of a main effect). So an effect of arousal related to pain and EBS combined with a ceiling effect could actually account for the whole pattern of effects, i.e., an increase in off-axis variability in high EBS_LBP at baseline, and an increase in variability around the primary axis alone in the complete group. The question then remains why presumably more subtle effects of between-group differences were observed for the off-axis movements and not for the primary axis movement. Here the previously offered explanations may have played a role.

To be more complete we have added the following information in the discussion section, r 71:

*We are not sure why these between-subject effects are observed for movement around the secondary axes. An increase in variability, only in the non-primary axes, with low back pain has also been observed in other studies (1,2). One might suggest that task constraints limit the scope to vary movements around the primary axis and an alternative explanation could be that an increased feedback gain (in the LBP group with high EBS) increases movement variability around the secondary axes more, because intrinsic stiffness is lower around these axes. However, the effect of threat was observed for movement around the primary axis, which would contradict both of these explanations.*

1. Ross GB, Mavor M, Brown SHM, Graham RB. The Effects of Experimentally Induced Low Back Pain on Spine Rotational Stiffness and Local Dynamic Stability. Ann Biomed Eng. 2015;43(9):2120–30.
2. Lamoth, C. J. C., Meijer, O. G., Daffertshofer, A., Wuisman, P. I. J. M., & Beek, P. J. (2006). Effects of chronic low back pain on trunk coordination and back muscle activity during walking: Changes in motor control. European Spine Journal, 15(1), 23–40. https://doi.org/10.1007/s00586-004-0825-y

2. For CycleSD, the interaction seems to be driven by the lower variability in the control subgroup with low EBS, while the high EBS control group has scores that are comparable to the LBP group, regardless of EBS. This does not seem to fit with the general statement that “… this interaction mainly reflected high variability in the LBP participants with high EBS.” (pg. 13) or that “… participants with LBP and high EBS, moved with higher variability, regardless of threat.” (pg. 14).

For CycleSD we agree with the reviewers that the interaction effect seems to be caused by a difference in CyclSD in the control group. To be more clear we have adjusted the sentence in the results section to:

Inspection of the data (Fig 4) showed that for spatial variability, this interaction mainly reflected high variability in the LBP participants with high EBS.

And in the discussion:

Additionally, participants with LBP and high EBS, moved with higher spatial variability, regardless of threat.

Other minor comments:
3. Abstract, last sentence:
“These results suggest that perceived postural threat may underlie changes in motor behavior in patients with LBP”. This conclusion does not seem appropriate, given the lack of between-groups differences in the data.

We have rephrased this to:

These results suggest that perceived postural threat may underlie changes in motor behavior. *Since LBP is likely to impose such a threat, this could be a driver of changes in motor behavior in patients with LBP, as is also supported by the higher spatial variability in the group with LBP and high EBS in the reference condition”.*

Introduction, second paragraph:
4. “Established risk factors for the development of chronic LBP (CLBP) are psychological factors …” could be interpreted as these being the only risk factors. I would suggest: “Established risk factors for the development of chronic LBP (CLBP) include psychological factors …”

We agree with the reviewer and have adjusted this sentence in the manuscript.

Methods:
5. Joint Kinematics – pg 8 - Were the marker data filtered after resampling the data to 100Hz. If not, why not? High frequency noise in these data can be amplified during the rigid body and Eular angle calculations.

After the resampling to 100 Hz the marker data were not filtered. We checked the effects of applying a filter (butterworth low pass filter, cut off frequency 10 Hz, order 2) for the ICC values in our previous study and the ICC values became lower (1). This is the reason we did not use a filter in our follow-up studies.

1. Wildenbeest MH, Kiers H, Tuijt M, van Dieën JH. Reliability of measures to characterize lumbar movement patterns, in repeated seated reaching, in a mixed group of participants with and without low-back pain: a test-retest, within- and between session. J Biomech. 2021;121:1–8. Available from: https://doi.org/10.1016/j.jbiomech.2021.110435

6. Variability - pg 8 - Was a cubic spline also used to normalize the angle data to 101 samples for analysis of spatial variability (as for the other two data interpolations mentioned in the methods)?

Yes, spline interpolation was used to normalize the angle data to 101 samples of spatial variability. To be more complete we have added this information to the sentence:

*For spatial variability, lumbar angle data for each cycle were normalized to 101 samples (0-100%) by spline interpolation for all three axes.*

7. Velocity variable - using s/repetition as a measure of velocity seems backwards to me. I would expect to see velocity as repetitions/s so that a lower velocity is reflected by a lower value.

Indeed, this is the inverse of your expectation. We have adjusted this in the methods section by adjusting the sentence in the ‘Amplitude and velocity’ paragraph:

*Velocity was calculated by dividing the number of repetitions by the total time of the trial.*

In the results section we have adjusted velocity in table 2 in (repetition/s) instead of (s/repetition).

8. Results:
Participants – first sentence “with respect to age, sex, length, …” should be “with respect to age, sex, height, …”

We agree with the reviewer and have adjusted this sentence in the manuscript.

9. Lumbar Movement Patterns (pg. 13) – for consistency “The MANOVA also revealed an interaction effect of LBP and EBS …” should be “The MANOVA also revealed an interaction effect of group and EBS …”

We agree with the reviewer and have adjusted this sentence in the manuscript.

10. Discussion:
Pg 14, second paragraph: “Possibly, arousal, in our study caused by pain and perceived threat, increases variability of muscle spindles afference …”. Given that the current study only manipulated threat as a variable, it would be better not to speculate on the role of pain in your findings. Alternatively, the fact that pain intensity was higher in the LBP/high EBS group should be referred to here (it is addressed on pg. 15).

We agree with the reviewer and have adjusted this paragraph in the manuscript. We chose for the alternative option to refer to the fact that pain intensity was higher in the LBP/high EBS group directly after the cited sentence.

Reviewer #2: Thank you for the opportunity to review this manuscript. The topic of negative pain-related cognitions as a factor driving altered movement patterns in individuals with persistent back pain is an important area of research. The authors clearly understand the difficulties with using clinical pain-related cognition questionnaires of trait characteristics to test the relationship between pain-related cognition and trunk movement. Further, they developed a novel protocol that presents a task-specific trunk postural threat and employed a task-specific measure attempting to capture the associated pain-related cognition. The following comments and suggestions are made with an eye toward strengthening the manuscript and the reader's ability to interpret their findings.

Intro:
1. In paragraph 3 of the introduction, you introduce “local dynamic stability”. However, it may not be clear to all readers clear what you are referring to. It appears this is a measure of motor control, but what is getting measured with this approach? Since you then go on to use it as a measure of the kinematics, it seems it deserves a little more in the introduction as to the specific construct of motor control you are measuring.

We agree with the reviewer and to clarify we have added the following information after the first sentence of this paragraph:

*Motor control is defined as the way in which the nervous system controls posture and movement to perform a specific task, including all motor, sensory and integrative processes (1). In this and our previous study (24) we used variability and local dynamic stability (LDS) as resp. linear and non-linear outcome measures to quantify the quality of this motor control. As described by Dingwell and Marin (2006), spatial variability is the mean standard deviation (MeanSD) of lumbar angles of a repeated movement (2). Temperal variability (CyclSD) is quantified as the standard deviation of cycle times (CycleSD) (5). As also described in our previous study (24), LDS is the inverse of how fast movement patterns are corrected, after very small perturbations, for example caused by eye blinking or breathing. LDS is commonly expressed by the local divergence exponent (LDE). The LDE is the mean logarithmic rate of divergence between neighboring trajectories in lumbar kinematics state space (3-7).*

1. J.H. Dieen van, P.N. Reeves, G. Kawchuk, L.R. Dillen van, P.W. Hodges, Motor control changes in low back pain: divergence in presentations and mechanisms, J. Orthop. Sports Phys. Ther. 49 (2019) 370–379, https://doi.org/10.2519/ jospt.2019.7917.
2. J.B. Dingwell, L.C. Marin, Kinematic variability and local dynamic stability of upper body motions when walking at different speeds, J. Biomech. 39 (2006) 444–452, <https://doi.org/10.1016/j.jbiomech.2004.12.014>.
3. S.M. Bruijn, O.G. Meijer, P.J. Beek, J.H. Van Dieen, Assessing the stability of human locomotion: a review of current measures, J. R. Soc. Interface 10 (2013), 20120999, <https://doi.org/10.1098/rsif.2012.0999>.
4. J.B. Dingwell, L.C. Marin, Kinematic variability and local dynamic stability of upper body motions when walking at different speeds, J. Biomech. 39 (2006) 444–452, <https://doi.org/10.1016/j.jbiomech.2004.12.014>.
5. N.R. Marques, C.Z. Hallal, D.H. Spinoso, M.H. Morcelli, L.F. Crozara, M. Gonçalves, Applying different mathematical variability methods to identify older fallers and non-fallers using gait variability data, Aging Clin. Exp. Res. 29 (2017) 473–481, https://doi.org/10.1007/s40520-016-0592-8.
6. R.B. Graham, E.M. Sadler, J.M. Stevenson, Local dynamic stability of trunk movements during the repetitive lifting of loads, Hum. Mov. Sci. 31 (2012) 592–603, <https://doi.org/10.1016/j.humov.2011.06.009>.
7. S. Mehdizadeh, A robust method to estimate the largest Lyapunov exponent of noisy signals: a revision to the Rosenstein’s algorithm, J. Biomech. (2019) 1–8, https://doi.org/10.1016/j.jbiomech.2019.01.013

2. “A contributing factor to these conflicting results could be that pain-related cognitions were measured with questionnaires addressing pain-related cognitions as a trait” – It would be helpful to the reader if you provided examples of which questionnaires you are referring to.

We agree with the reviewer and have added the following information:

A contributing factor to these conflicting results could be that pain-related cognitions were measured with questionnaires addressing pain-related cognitions as a trait *e.g. ‘the Pain Catastrophzing Scale’ (PCS) (1) or ‘the Pain Anxiety Symptoms Scale’ (PASS) (2).*

1. *Sullivan MJL, Bishop SR, Pivik J. The Pain Catastrophizing Scale: Development and Validation. Psychological Assissment. 1995;7(4):524–32.*
2. *McCracken LM, Zayfert C, Gross RT. The Pain Anxiety Symptoms Scale: development and validation of a scale to measure fear of pain. Pain. 1992;50:67–73.*
3. In paragraph 3 you introduce the need to use a task-specific measurement tool be used to measure pain-related cognitions. You then go on to describe your prior work where task demand “imposed a high back strain”. It may be best to use the term “imposed threat of pain or reinjury, until you get to the methods sections where you introduce the “Expected Back Stain” tool.

We agree with the reviewer and have adjusted this in the introduction*.*

In the methods we started the paragraph ‘Pain-related cognitions’ with the following sentence:

*To quantify the task-specific pain-related cognition, we established the expected back strain by asking “How demanding will this task be for your back?”. Participants responded to this question by means of an 11-points color-marked scale (0-10), based on the RPE-Borg scale, further on referred to as the ‘Expected Back Strain’ (EBS) (suppl. Material_1) (27).*

4. “We chose repeated seated reaching because this movement is a more demanding task for
the back than walking, which was the experimental task in most previous studies.” This statement needs a reference.

From a biomechanical point of view, forward reaching of the trunk creates a greater gravitational moment compared to walking and requires substantial muscle forces. Additionally, from clinical experience, substantial flexion is often considered as potentially pain provoking .

5. Methods:
Subjects: Were all the LBP subjects experiencing pain at the time of testing? If not, how might this have impacted the spread of data and your interpretation of findings?

Not all the LBP subjects experienced pain at the time of testing. We agree with the comment of the reviewer and that is why we discussed this issue in the discussion section:

“Despite the absence of correlations between lumbar movement patterns and patient-reported pain-intensity at the day of testing in the LBP-group, the pain-intensity was higher in the subgroup with high EBS (median 3 (IQR 1-4.5) compared to low EBS (median: 1 (IQR 0-2.72)) (U=61.5, p=0.03). Ross et al. (2015) demonstrated that nociception decreased local dynamic stability of trunk movement (10). So, although there is no direct correlation, nociception or pain may have been of influence on the between-subject effect through an increasing effect on variability and/or on the EBS-value.”

6. Did any of the back-healthy participants have a history of low back pain episodes?

Besides knowing that the participant had experienced more than one episode of non-specific LBP in the last 2 years, we did not ask for the history of low back pain episodes.

7. Kinematics- based on the location of the reflective markers, is the motion you derived just lumbar spine motion?

We measured the motion between a marker over the spinous process of the 8^th^ thoracic vertebra and over the 1^st^ sacral vertebra. So, the focus was the lumbar spine, which also has the largest range of motion in the flexion/extension direction, but part of the thoracic spine was included in the measurements.

8. EBS tool- Is this a measurement tool you developed and are there measurement properties established/published for the tool? The reference you use here does not support the measurement properties of this tool. What were the anchors for the scale or the descriptors used for the 0-10 levels? Given that you use this measure to represent pain-related cognition or task threat level and create groups based on participants' scores on the scale, its measurement properties and validated construct are important information for the reader.

We based the EBS on the 11-point color-marked Borg-Scale, because we wanted to use a simple measurement instrument to get a reliable impression of expected back strain without asking directly about pain. We chose a 0-10 scale, because this scale is more easy to respond to than the 6-20 level Borg Scale. To be more clear about this measurement we have added this measurement tool as supplementary material to the paper.

Because we changed the measurement tool by not asking about the perceived exertion but about the expected back strain, we can’t make any statements about measurement properties. As we described in our previous study (1) we did check for associations between:

1. EBS-values and PCS-scores: no correlations
2. EBS and PASS were moderately correlated: (Spearman correlation = 0.358, p ≤ 0.01).
3. (PASS-scores and PCS-scores in this study: strongly correlated (Spearman correlation = 0.753, p ≤ 0.01) )

1 Wildenbeest MH, Kiers H, Tuijt M, van Dieën JH. Associations of low-back pain and pain-related cognitions with lumbar movement patterns during repetitive seated reaching. Gait Posture. 2022;91:216–22

9. Does the cut score used, represent a real difference in perceived threat?

The cut score used is based on the median and is an arbitrary choice. We do not suggest that this reflects a hard difference between perceiving much or little threat. We used a median split because in this phase of research, we did not want to assume a linear relationship between the EBS-value and lumbar movement patterns. How to interpret this difference between high and low EBS is questionable. For this reason we have added the following paragraph to the discussion:

As in our previous study, we found an interaction effect of group and EBS on lumbar movement patters. This interaction may reflect different values attributed to EBS. In the subgroup LBP and high EBS, the experimental task may have activated pain-related cognitions, due to the increased risk for pain and injury, reflected in a higher EBS value, whereas an expectation of high strain may be more neutral to participants without LBP and the participants with LBP and low EBS. This indicates that, to assess task-specific perceived threat, additional questions may be needed concerning the value (threat, anxiety) attributed to the experimental task.

Additionally, we do see correlations with variability in the LBP-group. In the reference trial, in the LBP-group the EBS-scores are correlated to MeanSD_lateral-bending_ (r = 0.497, p ≤ 0.01), MeanSD_axial-rotation_ (r = 0.399, p ≤ 0.05) (1).

1. Wildenbeest MH, Kiers H, Tuijt M, van Dieën JH. Associations of low-back pain and pain-related cognitions with lumbar movement patterns during repetitive seated reaching. Gait Posture. 2022;91:216–22

10. Motor control is quantified by variability in cycle duration, motion variability, and slope of the divergence curves. Given that all of your readers may not be biomechanists or engineers, can you add a brief explanation of what aspects of motor control these variables represent?

We agree with this comment and have added the information as described in the response to your first comment.

11. Statistical analyses:
Please describe the statistical tests that were used to determine a lack of differences between the groups based on demographics.

We agree with the reviewer and have added the following information after the first sentence of this paragraph:

*Independent t-tests were used to test for differences between people with and without LBP, concerning height and weight. A Mann-Whitney U test was used to test for possible differences concerning pain-related cognitions between groups.*

In Table 1 we have added 2 columns on the right with the results of these statistical analyses.

In addition, because of the ordinal level of the EBS-scores, the statistical analysis to assess whether the task instruction affected the EBS-scores was adjusted to a Wilcoxon signed-rank test instead of a paired t-test.

12.The second sentence in the statistical analyses paragraph is difficult to follow.

We agree with the reviewer and have rephrased the sentence to:

*To assess whether the task instruction affected EBS, EBS-scores before the threat and reference conditions were compared using a Wilcoxon signed-rank test.*

13.It appears you set the p values at .05 for each analysis. Given that the dependent variables appear associated, can you address any concerns for type 1 errors within the discussion section?

We agree with the reviewer that it’s important to prevent type I errors and that is exactly the reason we choose for a mixed manova to test for the effect of perceived threat on the lumbar movement patterns, instead of separate paired sample t-tests for each dependent variable of movement patterns.

14. Results:
“Participants with and without LBP were comparable..” do you mean statistically not different?

Yes, we did mean that. Thank you for noticing: we’ve adjusted this sentence

15. The EBS dichotomized the groups, is the mean difference meaningful based on the descriptors you used for the scale. This should be addressed in the discussion.

See comment 9

Your figures 3 and 4 are well done. Thank you for sharing the full data with the reader.

16. Discussion:
The differences found between the reference and threat conditions do not numerically appear large, particularly when reviewing the figures. You have a previously published reliability stability on these variables which demonstrates moderate within-session reliability. This dataset should allow you to calculate minimal detectable differences which may assist in the further interpretation of the statistical differences you are reporting.

We chose in our previous study (1) to calculate the coefficient of variation (cv) in combination with ICC because we had multiple dependent variables with different measurement units. In addition, our goal was to conduct scientific research at the group level. Using these outcome measures on an individual level would be premature because we do not yet have reference values and additionally we do not know yet whether to correct for age or other possible confounders. If we want to use the outcome measures for diagnosis and evaluation over time at the individual level, then indeed it is necessary to calculate SEM. MeanSD seem to be most associated with pain cognitions, and at the same time this also is the outcome measure with a relatively large measurement error (compared to LDE). If the goal becomes to measure this outcome measure at the individual level, then in our opinion we should first work toward reducing this measurement error. For now, we did calculate the SEMconsistency and SDDconsistency for LDE and MeanSD:

|  | LDE | MeanSDflexion-extension | MeanSDaxial-rotation | MeanSDlateral-bending |
| --- | --- | --- | --- | --- |
| SEMconsistency (SDdiff/√2) | 0.18 | 0.32 degr | 0.15 degr | 0.18 degr |
| SDDconsistency (1.96 *SDdiff) | 0.51 | 0.88 | 0.41 | 0.51 |
| Mean change caused by threat | 0.1 (mean) | 0.37 (median) |  |  |
| Mean difference in LBP group between high low EBS |  |  | 0.2 (median) | 0.2 (median) |

Comparing the SDDconsistency of MeanSDflexion-extension and LDE with the significant change cause by threat measured on a group level in our current study, this change does not exceed the SDDconsistency used for individual diagnosis or responsiveness: resp. 0.1 and 0.37 degr.

1. Wildenbeest MH, Kiers H, Tuijt M, van Dieën JH. Reliability of measures to characterize lumbar movement patterns, in repeated seated reaching, in a mixed group of participants with and without low-back pain: a test-retest, within- and between session. J Biomech. 2021;121:1–8. Available from: https://doi.org/10.1016/j.jbiomech.2021.110435

17. Please expand upon the “neuromotor noise theory” and further relate your findings to the theory.

Van Galen’s neuromotor noise theory is a theoretical framework to explain a decreased motor precision in a condition of stress. A core tenet of the theory is that stochastic processes are present in the process of developing muscle force (neuromotor noise). Consequently, it’s impossible to move without spatial variability (kinematic noise). Stress (e.g., time pressure or a mental load) increases the neuromotor noise. A second core idea is that the filtering capabilities of the body reduce this noise. Such filtering is achieved through developing stiffness through co-contraction, which reduces kinematic variability. So, co-contraction can compensate for increased neuromotor noise to a certain level of stress where this noise can’t be compensated anymore and the motor precision and the performance of the movement decreases. (1).

We showed that there is a certain level of spatial variability in movement present during the reference trial. During the threat condition, the spatial variability increased. So in terms of the theory of van Galen (2000), during the threat condition, the signal-to-noise ratio exceeded the ability of the system to use stiffness (co-contraction) to compensate for the increased noise .

1. Galen G van, Huygevoort M van. Error, Stress and the Role of Neuromotor Noise in Space Oriented Behaviour. Biol Psychol. 2000;51:151–71.

To be more clear about this in the paper we have added the following paragraph to the discussion section:

*A core tenet of this theory of Van Galen et al., (2000) is that stochastic processes are present in the process of developing muscle force (neuromotor noise). Consequently, it is impossible to move without spatial variability (kinematic noise). This neuromotor noise is increased by stress (e.g., time pressure or a mental load) and co-contraction is a strategy to compensate for this increased neuromotor noise. Above a certain stress level, this neuromotor noise cannot be compensated anymore and the motor precision and the performance of the movement decrease. In terms of this neuromotor noise theory (1), during the threat condition, the signal-to-noise ratio exceeded the ability of the system to use stiffness (co-contraction) to compensate for the increased noise, reflected in an increased spatial variability.*

18. In the second paragraph: Does arousal equate to your EBS score?

Threat was used to increase arousal, but we assume that pain and inter-individual differences reflected in the EBS-score may also increase levels of arousal.

19. You mention that you believe arousal creates a change in variability mediated by muscle spindle afference and provided the reference that a previous study demonstrated “mental computation and fist clenching” increased variability of muscle spindle afference. Can your further explain or clarify how “mental computation and fist clenching” and dependence up internal attitude found in that study are similar to threat-related arousal?

To clarify this we have added the following paragraph to the discussion section:

*The results of Ribot showed that, in addition to the experimental task, individual characteristics influence the level of arousal or its effects. In some persons, the firing frequency of the muscle spindle afferents increased already while the instruction was given. Also, the duration of the increased sensitivity of the muscle spindles showed interindividual differences, varying form a decrease in sensitivity immediately after ending the experimental task to a continuing of this increased sensitivity for several minutes .*

Ribot-Ciscar E, Rossi-Durand C, Roll JP. Increased muscle spindle sensitivity to movement during reinforcement manoeuvres in relaxed human subjects. Journal of Physiology. 2000;523(1):271–82.

20. “Possibly, arousal, in our study caused by pain and perceived threat, increases variability of muscle spindles afference, which could then account for the effects on lumbar movement patterns observed here” I may have missed it, was a change in pain measured prior to the reference trial?

There was no change in pain measured prior to the trial. The word pain in this sentence refers to pain-intensity measured at the day of testing.

To be more clear we have /adjusted this sentence into:

“Possibly, arousal, in our study caused by pain*-intensity measured at the day of testing*, and perceived threat, increases variability of muscle spindles afference, which could then account for the effects on lumbar movement patterns observed here”

21. The sentence “We speculate that higher… local dynamic stability” is long and difficult to follow. It may help to break it apart.

We agree with the reviewer and have changed this sentence into 2 sentences:

*We speculate that higher muscle spindle reflex gains, observed in patients with LBP and especially in those with negative pain-related cognitions are effective in dealing with external perturbations (29). At the same time higher muscle spindle reflex gains may amplify noise in spindle inputs (local muscle strain and strain rate) and as a consequence increase movement variability and decrease local dynamic stability.*

22. “This is in line with the conclusion of Saito et al. that linear measures reflecting the magnitude of the variance are more sensitive to pain-related cognitions than non-linear measures reflecting the structure of the variance (31).” Could you expand more as to why this might be the case?

To expand we have added the following information in the paper after the cited sentence:

*A possible explanation could be that LDE is a more reliable measurement compared to MeanSD, but less sensitive as demonstrated in our previous study (1). LDE had a smaller coefficient of variation (cv) than MeanSD. At the same time, the within-session intraclass correlation coefficient (ICC) of LDE was lower than that of MeanSDflexion-extension. This implies that the variance of the LDE between participants was lower compared to that of MeanSDflexion-extension. This may be reflected in a lower responsiveness to threat, which caused an increase of 3% in LDE and 22% in MeanSDflexion-extension.*

1. M.H. Wildenbeest, H. Kiers, M. Tuijt, J.H. van Die¨en, Reliability of measures to characterize lumbar movement patterns, in repeated seated reaching, in a mixed group of participants with and without low-back pain: a test-retest, within- and between session, J. Biomech. 121 (2021) 1–8, https://doi.org/10.1016/j. jbiomech.2021.110435.
