# Supplementary material for: Effect of postural threat on motor control in people with and without low back pain
Source: PLoS One. 2023 Mar 27;18(3):e0280607. doi: 10.1371/journal.pone.0280607 (PMC10042370; doi:10.1371/journal.pone.0280607)
Supplement: Attachment — Submitted filename: Response to Reviewers_revision_2.docx [file pone.0280607.s003.docx]

Dear Reviewers,

Thank you for your constructive comments. The changes made to the manuscript in response to your comments have been copied in the rebuttal below.

Yours sincerely,

Meta (M.H.) Wildenbeest

6. Review Comments to the Author

1) Page 3, line 72 – I would not abbreviate ‘resp.’. I assume that this should read: “… variability and local dynamic stability (LDS) as, respectively, linear and non-linear outcome measures …”

We agree with the reviewer and have adjusted this sentence in the manuscript.

2) Page 3, line 74 – ‘Temperal variability’ should read ‘Temporal variability’

We agree with the reviewer and have adjusted this sentence in the manuscript.

3) Page 4, line 77 - ‘… after infinitesimally perturbations …’ I assume should be ‘… after infinitesimally small perturbations …’. I would, however, suggest that ‘infinitesimally’ may be an exaggeration and that ‘… after very small perturbations …’ would be more appropriate.

We agree with the reviewer and have adjusted this sentence in the manuscript.

4) Page 4, line 85 – I am not sure that pain-related cognitions can be considered to be ‘character traits’. I would suggest writing simply “… pain-related cognitions are dynamic and mutable, influenced by the task at hand and its current context.”

We agree with the reviewer and have adjusted this sentence in the manuscript.

5) Page 6, line 139 (and elsewhere throughout the manuscript) – I believe that both ‘T’s and the ‘B’ should be capitalized when referring to the ‘STarT Back’ screening tool. The acronym used on the same line (SBST) also appears as (STBST) elsewhere in the manuscript.

We agree with the reviewer and have adjusted this sentence in the manuscript.

6) Page 6, line 142 – both uses of ‘indicated’ do not appear to fit the context of the sentence. I would suggest either “… with a minimum score indicating …” or “… for which a minimum score indicated …”).

We agree with the reviewer and have adjusted this sentence in the manuscript.

7) Page 15, line 47 – at the end of the line ‘… varying form a …’ should read ‘… varying from a …’

We agree with the reviewer and have adjusted this sentence in the manuscript.

8) Page 17, lines 101-103 – I would suggest that the more relevant measurement property be emphasized. For example, "A possible explanation could be that LDE is a less sensitive measure than MeanSD, despite being more reliable, as demonstrated in our previous study."

We agree with the reviewer and have adjusted this sentence in the manuscript.

9) Page 17, lines 103 – the acronym for ‘coefficient of variation’ is written in lowercase (cv) while all other acronyms are in uppercase. I would suggest using (CV) throughout.

We agree with the reviewer and have adjusted this sentence in the manuscript.

10) Throughout the manuscript, the use of commas does not seem to follow any convention that I am familiar with. Given that more than one convention exists, however, I would suggest that the editor (or copy editor) suggest any changes that might be required based on the guidelines of the journal.

Reviewer #2: Thank you for addressing our prior comments.

We would like to make an additional suggestion. Since you acknowledge the limitations of the EBS instrument, "The cut score used is based on the median and is an arbitrary choice. We do not suggest that this reflects a hard difference between perceiving much or little threat." It would make it more transparent to the reader that you are not suggesting the high vs. low EBS reflects a meaningful difference in perceived threat if you acknowledge this as a point to be considered when interpreting the significant interaction reported for LBP and High EBS related to spatial variability.

We agree with the reviewer and have added the following sentence to the discussion section (line 357) in the manuscript:

*…….although we must be cautious in interpreting high and low EBS as a meaningful difference in perceived threat, given the lack of published measurement properties of the EBS-tool.*

In addition, we have adjusted the conclusion (line 404):

Postural threat resulted in increased variability and decreased stability of lumbar movement patterns, independent of the presence of low back pain (LBP) or level of ‘Expected Back Strain’ (EBS). Among participants with LBP, those with a higher level of task-specific EBS showed higher spatial variability of lumbar movement patterns, regardless of postural threat. These results suggest that perceived postural threat may underlie changes in motor behavior due to LBP.

In addition, we have adjusted the conclusion in the abstract (line 39):

….., as also supported by the higher spatial variability in the group with LBP and higher EBS in the reference condition.
